# Development of a Predictive Model for Iron Levels in Bovine Muscle Tissue Using Hair as a Predictor

**DOI:** 10.3390/ani14071028

**Published:** 2024-03-28

**Authors:** Kirill Narozhnykh

**Affiliations:** Department of Veterinary Genetics and Biotechnology, Institute of Veterinary Medicine and Biotechnology, Novosibirsk State Agricultural University, 160 Dobrolyubova Str., Novosibirsk 630039, Russia; k@narozhnykh.ru

**Keywords:** large ruminants, Hereford breed, iron, modeling, regression, hair, atomic absorption analysis

## Abstract

**Simple Summary:**

This study focuses on developing a noninvasive model to predict iron content in Hereford cattle muscle tissue, a critical factor in both animal health and meat quality, relevant to sustainable livestock management. Conducted in the Novosibirsk region (Russia) in 2023, the research employed atomic absorption analysis on muscle tissue and hair samples from cattle. The construction of a regression model using the least squares method was pivotal in identifying the most effective predictors, including magnesium, potassium, iron, aluminum, and chromium levels in hair. The outcomes of this study have broad implications for both ecology and veterinary medicine, particularly in the assessment of ecological well-being and managing the iron load in animals.

**Abstract:**

The assessment of iron levels in cattle muscle tissue is crucial for livestock management because it influences both animal health and meat quality, key factors in sustainable development. This study aimed to develop an optimal model for noninvasively predicting the iron content in Hereford cattle muscle tissue, contributing to a comprehensive understanding of the animals’ elemental status. The research involved the atomic absorption analysis of muscle tissue and hair samples from cattle. A regression model was constructed using the least squares method to identify the most effective approach. These findings have ecological applications, aiding in evaluating environmental health and establishing acceptable iron thresholds for animals. The proposed mathematical model utilizing biomarkers (levels of Mg, K, Fe, Al, Cr in hair) will allow for the assessment of iron levels in cattle muscle tissue throughout the period of productive use, with the possibility of adjustment and tracking the changes in elemental status over time. The utilization of the developed method will enable the diagnosis of animal elementosis and assessment of the iron level burden. Subsequently, this will allow for the improvement of the qualitative characteristics of the final product. Thus, the obtained data contribute to fundamental knowledge regarding the content and variability of iron levels in the muscle tissue of cattle.

## 1. Introduction

The iron content in cattle muscle tissue is a significant factor in understanding and managing both the health of cattle and the quality of beef produced for consumption. Detecting iron content in the muscle tissue of live cattle typically involves methods that are either noninvasive or minimally invasive, providing a snapshot of the animal’s overall iron status. Blood sample collection and analysis are the most prevalent approaches. This method assesses iron status by measuring parameters such as serum iron, ferritin, and total iron-binding capacity [1]. While effective, it has its own set of advantages and limitations. Recently, advanced research has shifted toward employing predictive models. These models estimate iron content by considering various factors, including breed, age, diet, and known absorption rates, offering a comprehensive and nuanced understanding of iron content in cattle.

In cattle, alterations in iron homeostasis parameters may occur due to various diseases. For instance, mastitis or parasitic invasions (such as ankylostomiasis) can lead to inflammation, blood loss, and changes in iron metabolism in the animal’s body [2,3]. Chronic conditions, such as chronic stress or prolonged periods of poor nutrition, can also affect the iron homeostasis markers in cattle [4].

Disturbances in iron homeostasis can be reflected in various morphological parameters of blood. Iron deficiency can lead to anemia, which may manifest as a reduction in the hemoglobin (Hb) levels in the blood. Iron deficiency can affect the quantity and shape of red blood cells (RBCs), for instance, resulting in microcytosis (reduced erythrocyte size) and hypochromia (the reduced hemoglobin content in erythrocytes). Additionally, this is accompanied by decreases in Mean Corpuscular Hemoglobin (MCH) and Mean Corpuscular Hemoglobin Concentration (MCHC) [5,6,7,8]. The determination of transferrin (TF) levels is used in the differential diagnosis of iron-deficiency anemias, characterized by decreased serum iron content, an increase in the level of this glycoprotein, and consequently, a decrease in the percentage of transferrin saturation with iron [9]. However, in cases of iron overload in the body, the unsaturated iron-binding capacity (UIBC) decreases. Therefore, to assess the blood’s ability to bind iron to transferrin, a laboratory test called total iron-binding capacity (TIBC) is utilized [10]. The analysis of a soluble transferrin receptor (sTfR) concentration in blood is utilized for investigating iron deficiency anemia and assessing iron the functional status. Due to its insensitivity to inflammatory processes, sTfR can detect anemia in animals already suffering from inflammatory conditions, and it holds particular significance in distinguishing between anemia in chronic conditions and anemia caused by inadequate iron intake [11,12]. However, such markers indicate iron deficiency in the overall animal organism and do not provide information about the quantitative level of iron in animal muscle tissue. The method proposed by us will allow for the assessment of iron levels in muscle tissue and, if necessary, its monitoring throughout the animals’ productive use.

The iron content in the muscle tissue of cattle can vary depending on various stochastic and fixed factors, such as the animal’s age, sex, diet, living conditions, breed, and other related aspects [6,13,14]. In this regard, the average iron content in the skeletal muscle tissue of large ruminants varies widely, ranging from 10 to 50 mg/kg [15,16,17,18,19,20,21,22,23,24,25,26,27,28]; in earlier studies, iron concentrations of up to 54 mg/kg were found [29]. A tendency can be noted that, in European studies, the level of zinc in the muscle tissue of animals was typically below 40 mg/kg [15,17,18,19,20,21,22,23,24,30], except in Slovakia, where samples of muscle tissue from animals raised in the vicinity of the metallurgical plant were studied [25]. Higher metal concentration levels in skeletal musculature were observed in animals from countries with developing economies [26,27,28].

Approximately 5–15% of the iron ingested through feed is typically absorbed, yet in cases of deficiency, the absorption process can double [31]. A study conducted by Knowles et al. [32] demonstrated that the rate of iron reabsorption is inversely proportional to the ferritin level in serum. Iron absorption from the gastrointestinal tract depends on endogenous factors such as age, body iron levels, the gastrointestinal tract environment, and overall health status. Exogenous factors such as chemical form, iron quantity, and other feed components also influence the iron absorption in the intestine, contributing to its increase or decrease. In the in vitro study, it was found that the addition of ascorbic acid increases the absorption of iron from sodium caseinate–ferric iron and ferrous sulfate to a similar level, which significantly surpasses the absorption from iron pyrophosphate [33]. Phosphorus-containing compounds may be present in the diet of cattle, especially if it includes grain feeds. Phytates and tannins are capable of forming insoluble complexes with iron, thereby hindering its absorption in the cattle’s intestines [13]. Following erythrocyte breakdown, a significant portion of iron is reabsorbed and utilized for synthesizing new hemoglobin. Erythrophagocytosis occurs in the spleen, liver, and bone marrow. These organs contain siderophages, which phagocytose and degrade old or damaged erythrocytes. The products of erythrocyte degradation, such as hemoglobin, iron, and bilirubin, are then processed and recirculated for further utilization or excretion from the body. Residual iron that has not been absorbed is excreted from the body in feces [6,33,34,35,36,37].

This study is a continuation of exploratory research aimed at developing models for assessing iron levels in muscle tissue.

The models presented in the scientific literature are constantly being improved, providing more accurate and efficient estimates. To understand the focus of this study, we present the key approaches to assessing iron in animal muscle tissue.

Near-infrared spectroscopy (NIRS): NIRS is a nondestructive technique that uses the absorption of near-infrared light by molecules in muscle tissue to estimate the iron content. It is widely used in the livestock industry due to its speed and ease of use. MRI is a noninvasive imaging technique that can provide detailed information about the composition of muscle tissue, including iron content. It offers high-resolution images and is useful for research purposes. Muscle biopsies are commonly used to directly measure iron levels in muscle tissue. These samples can then be subjected to chemical analysis techniques such as atomic absorption spectrometry or inductively coupled plasma–mass spectrometry (ICP–MS) to accurately determine iron concentrations. Ultrasound is used to assess muscle characteristics, including muscle density and fat content. Iron levels cannot be directly measured; however, they can provide valuable information related to the overall quality of muscle tissue. Machine learning models, including artificial neural networks and regression models, can be trained on datasets containing various muscle tissue characteristics, including iron levels. These models can then predict the iron content in muscle tissue based on other measurable parameters, such as age, weight, and breed. Researchers have also explored the use of biochemical markers in blood samples to indirectly assess iron levels in muscle tissue. These markers include serum iron, ferritin, and transferrin saturation levels. Genetic markers associated with iron metabolism and muscle iron content have been identified in cattle. These markers can be used for genetic selection in breeding programs to produce cattle with desired iron levels in their muscle tissue. DXA is primarily used in human medicine but has also been applied to assess the composition of meat samples, including iron content. Moreover, these findings can provide valuable insights into muscle tissue composition.

The choice of model or technique for assessing iron in the muscle tissue of cattle often depends on various factors, such as cost, accessibility, accuracy, and the specific goals of the assessment. Researchers continue to refine these models and explore new technologies to increase the accuracy and efficiency of iron assessments in cattle. These advancements ultimately contributed to improved livestock management and meat quality.

Each method has its own set of advantages and disadvantages. Therefore, providing specialists with more opportunities to assess gland levels in animals can lead to a more effective meat quality. Consequently, our study focused on determining the concentrations of trace elements in biosubstrates. This method assesses the levels of these genes within an organism. Serum blood, hair, and other substances are commonly used as diagnostic indicators [38,39]. The mineral contents of biosubstrates differ, which can impact their ability to determine an organism’s elemental status. A drawback of studying mineral substances in serum and blood plasma is that a deficit in these elements appears after the patient becomes symptomatic due to the body’s depletion linked to increased excretion. Therefore, specific changes in the concentrations of individual elements often cannot be detected in a timely manner, and these fluctuations fall within the margins of the error of the analysis method [40,41,42].

Due to the high informativeness of hair in studying the elemental profile, the findings of this research have found broad applications in hygiene, toxicology, and medical investigations, particularly in identifying cases of poisoning by toxic elements [41,42,43,44,45].

The primary objective of the present research was to identify an optimal and efficient predictive model for iron levels in the muscle tissue of large ruminants in the Hereford breed. This model aims to assess the animals’ elemental status noninvasively during their lifetime.

## 2. Materials and Methods

### 2.1. Ethics Statement

The animals were kept under standard conditions of an industrial complex, complying with veterinary and zootechnical requirements in accordance with legislation [46,47] and under standard conditions specific to each species and breed. Feeding was carried out using standard complete compound feed, taking into account the animals’ age, body weight, and productivity. Drinking water for the animals was sourced from local utility and drinking sources, meeting hygiene requirements [48,49].

Animal slaughter was conducted in a commercial abattoir in accordance with applicable requirements, technological instructions, and regulatory documents [50,51].

### 2.2. Experimental Design

This study was conducted in 2023 on animals (*n* = 31) of the Hereford breed raised in the southern region of Western Siberia (Russia).

The sample size was determined for ethical and economic reasons. Conducting research involving the slaughter of farm animals is challenging due to difficulties in accessing and limited resources for data collection. The age of the animals at the time of slaughter was 16–18 months. The animals were raised on a farm located in the south of the Novosibirsk region in the Maslyaninsky district, Russia (coordinates 54°32′45.1″ N 84°13′04.1″ E or 54.545862, 84.217812). The animals were kept on free-range pasture in an ecologically safe area more than 100 km away from industrial enterprises and large cities. The ages of the young bulls is determined by the fact that at 16–18 months, they reached optimal weight, and their physiological growth ends, making it the most economically advantageous time for slaughter.

To search for a model predicting iron levels in the muscle tissue of Hereford cattle, a set of predictors was utilized and subsequently renamed for convenience according to Table 1. The distribution of the studied characteristics deviated from Gaussian distribution; therefore, to assess the content of each element in the samples under investigation, the median as well as the values of the first and third quartiles (Q1–Q3) were calculated.

Preslaughter health assessments indicate that all the animals were clinically healthy. Rectal thermometry was used to measure the body temperature, which ranged from 37.5 to 39 °C. All animals were fasted for at least 12 h before slaughter and had unrestricted access to water. Samples of skeletal muscle weighing 100 g were taken from the *m. obliquus externus abdominis*. Muscle tissue and hair samples were collected immediately after slaughter. The selected muscle tissue samples were cooled to 4 °C and dispatched to the laboratory, where they were stored at a temperature of −24 °C until analysis was conducted. Hair samples weighing 10 g for atomic absorption analysis were collected from the withers area. The hair length ranged from 1 to 4 cm. The hair samples were packed in envelopes made of heavy-duty paper and stored frozen, similarly to the muscle tissue.

### 2.3. Atomic Absorption Analysis

For atomic absorption analysis, a 10 g hair sample was weighed. To clean the hair from impurities, the sample was placed in a flask with distilled water and then mixed for 1 min using a mixer at a rotating speed of 225 RCF. The water was then changed 10 times, repeating this procedure. Subsequently, the hair sample was dyed with acetone (CAS: 67-64-1, XILONG, Shantou, China) and left for 2 min, after which the remaining solution residues were rinsed 3 times with deionized water and dried at room temperature. Then, the hair samples were dissolved in 2 mL of nitric acid (CAS 7697-37-2, XILONG, Shantou, China) and placed in a standard autoclave in the microwave oven MARS-5 (CEM). The autoclave was gradually sealed over 40 min, after which the temperature was increased to 180 °C to perform the dissolution. The resulting solution was transferred to a volumetric flask. The solutions were analyzed after 10- and 100-fold dilutions using calibration solutions prepared based on multielement standards.

The preparation of internal muscle tissue samples for atomic absorption analysis proceeded in the following sequence: the vessel was washed in a soapy solution, rinsed with tap water and then rinsed with bidistilled water before drying. A sample from the test (100 g) was ground using the analytical mill IKA A11 basic and homogenized with an IKA Ultra Turrax Tube Drive control Disperser (IKA-Werke GmbH & Co. KG, Staufen im Breisgau, Germany) until a homogeneous mass was obtained. Subsequently, it was dried in an oven at a temperature of 60–70 °C for approximately 12 h until a constant mass was achieved. From the obtained dry residue, 3 g was weighed and ashed in a muffle electric furnace EKPS 10 (Code 4009) (Smolensk SCTB SPU, Smolensk, Russia) at a temperature of 500–550 °C. After 10–15 h, the mineralization process was completed, and the ash acquired a gray or white color. After cooling to room temperature, the ash residue was dissolved in 3 mL of 50% hydrochloric acid (CAS 7647-01-0, XILONG, Shantou, China) and then dried on a hotplate. This residue was transferred to a volumetric flask and diluted with 25 mL of distilled water [52]. The concentration of iron in the resulting solution was determined at an analytical wavelength of 510 nm using an analyzer.

The atomic absorption analysis of muscle tissue was conducted using an MGA-1000 spectrometer (Lumex LLC, Saint Petersburg, Russia). The measurements of the chemical element levels in the hair were conducted using the iCAP-6500 spectrometer (Thermo Scientific, Waltham, MA, USA). In the muscle tissue, the concentration of iron was determined, while in the hair, the levels of several heavy metals were determined: P, Ca, Mg, Na, K, Fe, Mn, Cu, Zn, Al, Ba, and Cr. 

### 2.4. Statistical Analysis

Checking the assumptions typical for regression analysis was conducted following the protocol for data exploration to avoid common statistical problems [53]. Outlier testing for the original data was performed using the Grubbs test [54]. The assessment of residual distribution normality was executed using the Shapiro–Wilk method [55]. The detection of related variability between features was carried out using the Spearman correlation coefficient [56]. The assessment of multicollinearity was performed by calculating the variance inflation factor for each parameter [57]. The model coefficients were calculated using the method of least squares. Studentized residuals with high Cook’s distance values were analyzed for outliers using Bonferroni correction [58].

Statistical analysis and visualization of the original datasets were conducted using the R statistical programming language and RStudio development environment.

The use of exploratory analysis in the study was necessary for fitting regression models and selecting a pool of predictors. Initially, an assessment of multicollinearity was conducted. Its presence may render model coefficient estimates unstable, making it challenging to discern the individual contributions of factors to the variance of the response variable. This situation may paradoxically lead to regression model coefficients being statistically insignificant, while the model as a whole remains statistically significant based on the Fisher criterion. Therefore, to assess the associations between variables, Spearman’s correlation coefficients were calculated, and correlation matrices and scatterplots were constructed. Model fitting in the study began with the creation of a full model across all the subsets. Then, as a result of model selection based on internal quality criteria, starting with the full model across all the subsets, two candidate models were selected to check assumptions regarding residuals. To ensure the validity of the models for assessment, the final stage of the study involved checking the assumptions regarding the residuals of the selected model. As multiple regression models are specific examples of general linear models, assumptions regarding residuals align with the Gauss–Markov theorem conditions.

## 3. Results and Discussion

### 3.1. Exploratory Analysis

**Figure 1:**
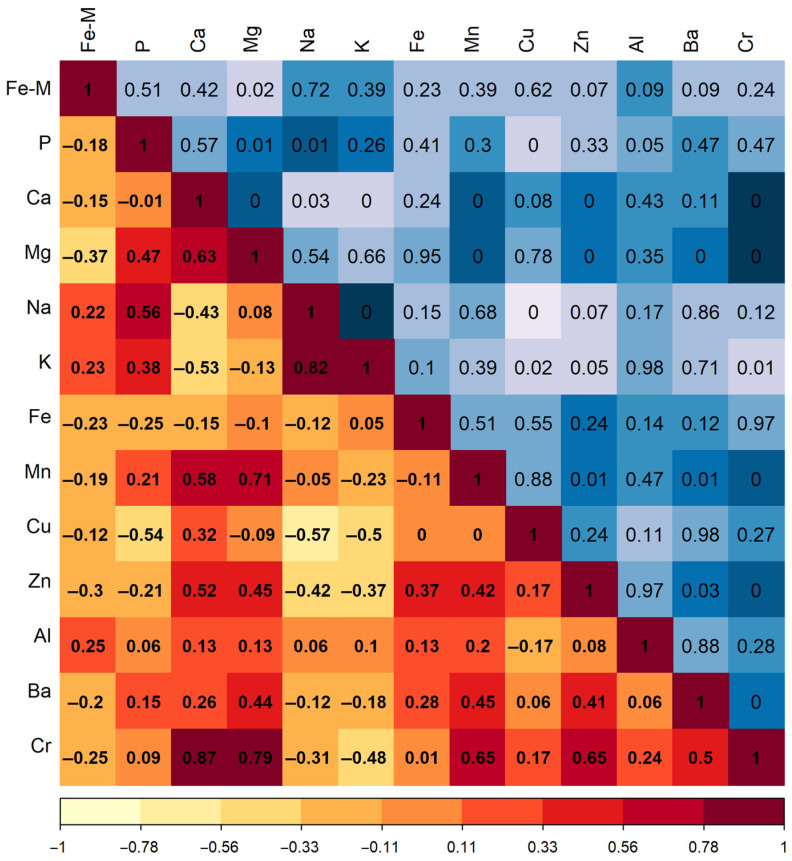
Correlation matrix of variables in the regression models. Fe-M represents the iron content in the hair. The other chemical element designations pertain to their levels in the hair. The displayed correlation matrix indicates sufficiently high multicollinearity among the levels of chemical elements in the hair. In the lower triangle on a red background are the computed Spearman correlation coefficients, while in the upper triangle (on a blue background) are the levels of significance for these coefficients.

The computed Spearman correlation coefficients are shown in the lower triangle (with a red background), while the significance levels are shown for these coefficients in the upper triangle (with a blue background) (Figure 1). The analysis revealed a considerable number of relationships between the variables. However, the dependent variable is associated with the level of magnesium in hair, which, in turn, is correlated with the concentration of manganese, zinc, barium, and chromium. Within this pool of potential predictors, linked variability has been identified, for example, between the content of zinc and that of barium and chromium. Including all of these variables in the model will result in multicollinearity effects. Hence, when selecting the optimal model for forecasting iron levels in muscle tissue, it is essential to choose a combination of coefficients that ensures a minimal value of the variance inflation factor.

### 3.2. Model Fitting

The model with the highest adjusted coefficient of determination included six predictors (Table 2). 

The assessment of the statistical significance of the coefficients and candidate models as a whole is presented in Table 2 and Table 3. In general, all the models were significant; however, the compact model with five coefficients exhibited the highest F-criterion value. Additionally, in this model, each coefficient was significantly different from that of the other two candidate models (Table 2 and Table 3).

**Figure 2:**
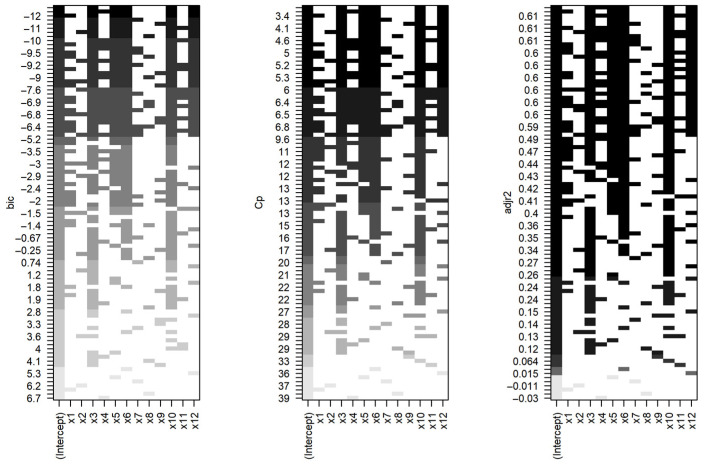
Ranking of models for predicting the level of iron in muscle tissue based on the Bayesian information criterion, Mallows criterion, and adjusted coefficient of determination (from **left** to **right**). The best models are ranked from **bottom** to **top** based on three predictors. The optimal combination of coefficients is located at the top of the figure. According to the Bayesian information criterion and the Mallows criterion, the best model contains the same predictors. The coefficients of the models for internal quality criteria are indicated in black. If the color is white, it means the coefficient is absent in the model.

The second candidate model was more concise, consisting of five variables, and demonstrated superior values according to the Bayesian information criterion, Akaike criterion, and Mallows criterion (Table 3 and Table 4, Figure 2).

The assessment of the variance inflation factor (VIF) for candidate models reveals that only the model with five predictors exhibits the lowest multicollinearity (Table 5). In this model, only two coefficients (x3 and x12) had increased VIF values, indicating a minor correlation between these variables and the others. In contrast, half of the coefficients in the other models displayed high VIF values, indicating instability in the predictive results of such models.

**Figure 3:**
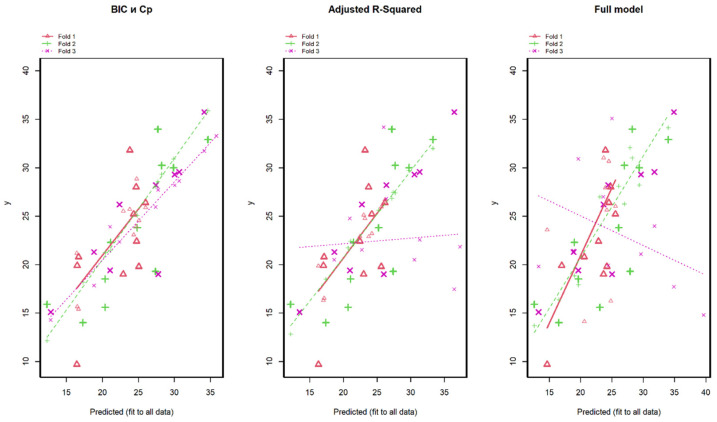
Visualization of the candidate models for forecasting iron levels in muscles using cross-validation with partitioning into 3 blocks. The model selected by BIC and Cp Mallows provides the most accurate estimates of iron levels in muscle tissue during cross-validation.

To assess the forecasting effectiveness of each candidate model, external quality criteria need to be employed. Therefore, to determine the best model, we conducted cross-validation by partitioning the observations into three blocks. Based on the analysis results, the best fit was observed for the model with five predictors (Figure 3, first from the left), where it is clearly visible that the regression line slope was maintained.

This observation is confirmed by calculating the mean square using the cross-validation method. Moreover, this method allows the unbiased estimates of the coefficient of determination to be obtained (Table 6). As a result, all the external assessments of model quality indicate that the model with five predictors (x3, x5, x6, x10, and x12) is optimal and best suited for predicting iron levels in the muscle tissue of Hereford cattle.

### 3.3. Assessing Residual Assumptions

**Figure 4:**
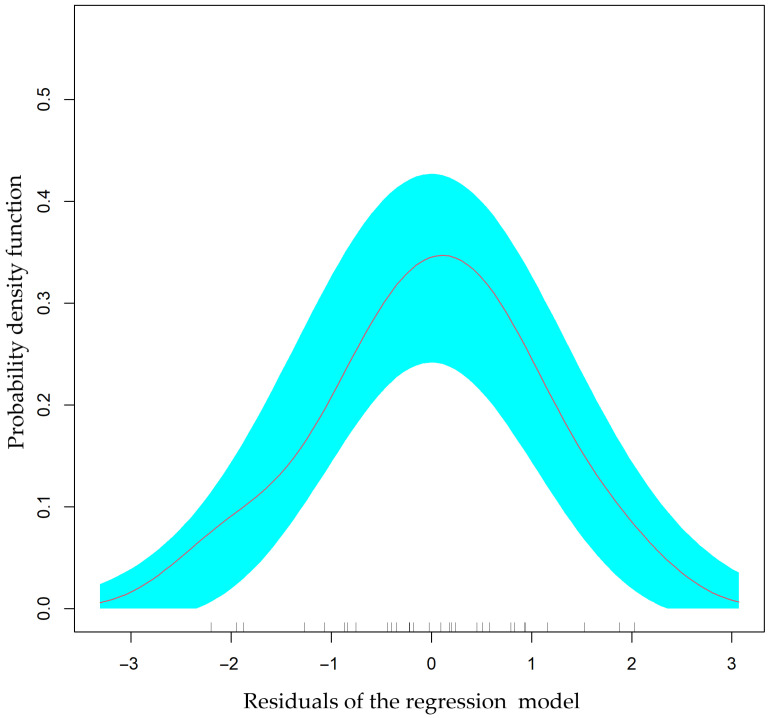
Distribution of residuals in the regression model estimating iron levels (mg/kg) in muscle tissue. The distribution of model residuals corresponds to the Gaussian distribution, indicating the reliability of the model. The distribution of model residuals is indicated by the red line. Confidence intervals of the normal distribution are depicted in blue.

**Figure 5:**
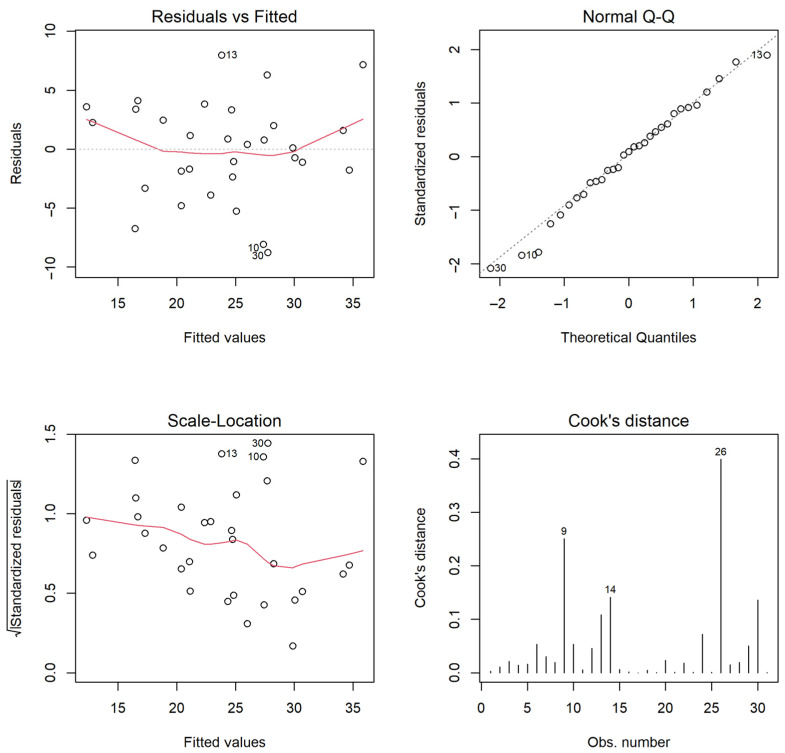
From **left** to **right**: residuals versus fitted values, quantile–quantile plot, square root of standardized residuals versus fitted values, and Cook’s distances. The diagnostic plots indicate that the selected model complies with the assumptions imposed on linear regression. The numbers on the figure represent options that could potentially be outliers or points of high intensity.

Initial assessments involved testing the residual distribution for compliance with a normal distribution using formal Anderson–Darling (A = 0.16; *p* = 0.93) and Shapiro–Wilk (W = 0.98; *p* = 0.90) tests. The visual inspection of the residual distribution also confirmed the adherence to the Gaussian distribution assumption (Figure 4 and Figure 5, top right).

**Figure 6:**
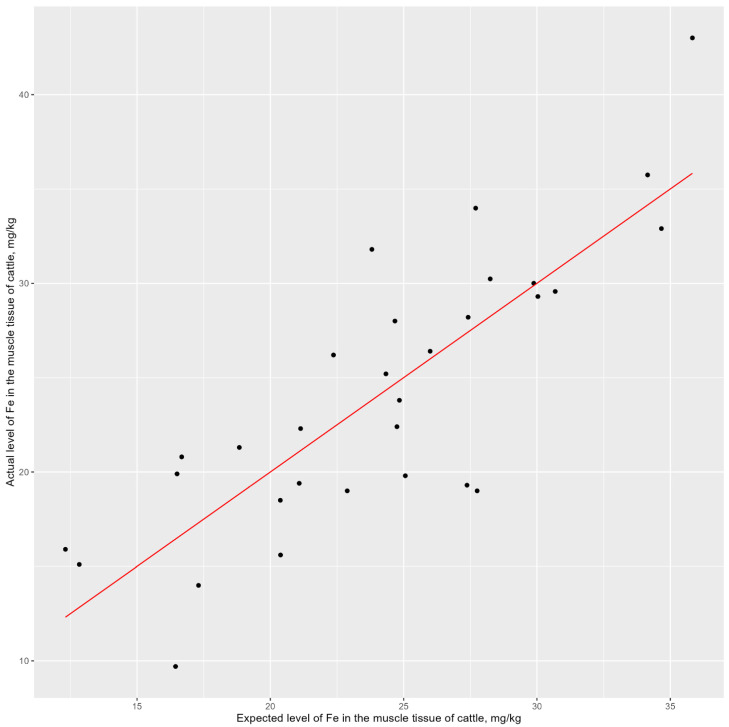
Model of the expected and actual iron levels in the muscle tissue of cattle. The proposed model adequately describes the value of the dependent variable. The red line represents the trend line for the iron level prediction model in muscle tissue. The intersections of expected and actual levels of Fe in the muscle tissue of cattle are marked by dots.

The spread of residuals and the square root of standardized residuals against predicted values indicate the homoscedasticity of residual variance (Figure 6, bottom left and top graphs). The bottom right graph of Figure 6 is intended to identify influential observations. The ordinal numbers represent observations with high Cook’s distances. These observations could be outliers. Through a formal test of residuals with Bonferroni correction, the Studentized residual with the maximum value is checked against the t distribution. Consequently, the maximum value of the Studentized residual was 2.92, corresponding to a corrected significance level (*p*) of 0.06. Therefore, there is no ground for considering potentially influential observations as outliers.

Thus, to establish the best and most accurate model for predicting the iron concentration in muscle tissue, it is necessary to determine the levels of magnesium, potassium, iron, aluminum, and chromium in hair (mg/kg) and substitute these values into the regression equation:

y = 25.862 − 0.043 × Mg + 0.008 × K − 0.214 × Fe + 0.235 × Al + 1.904 × Cr,(1)

where y represents the concentration of iron in the muscle tissue (mg/kg).

A scatter plot of the predicted and observed values with a trend line is presented in Figure 6. The obtained model demonstrated a reasonably high level of accuracy based on external quality evaluation criteria (Table 6, Figure 3). Therefore, the NRS-2002 can be recommended for predicting the level of iron in muscle tissue from Hereford cattle.

When comparing our current model with the previously proposed model for predicting the level of iron in muscle tissue, which utilizes biochemical blood parameters as predictors [59], both models exhibit strengths and weaknesses. The model proposed earlier is more compact, devoid of multicollinearity in all coefficients, and has a slightly greater coefficient of determination. However, a drawback of this model is the invasive procedure required for sample collection in blood analysis. On the other hand, biochemical analysis is less expensive than atomic absorption hair analysis. Nonetheless, each of the proposed models may be relevant depending on the context of their application. Overall, the two methods demonstrate comparable accuracy; thus, their choice will depend on the conditions of animal maintenance and the feasibility of analyzing the original samples.

In turn, the elemental analysis of hair offers several advantages: the collection of hair samples for analysis is extremely simple and nontraumatic; samples do not require specialized equipment for storage and transportation; hair can be stored for almost an indefinite period without losing its informational value; the concentration of most chemical elements in hair is greater than that in physiological fluids traditionally used for clinical and biochemical analyses, allowing for a significant expansion of the available chemical elements for analytical determination; additionally, hair analysis represents integrative information, reflecting the averaged state of biochemical processes during the period of hair formation (growth), thus significantly mitigating the influence of short-term factors [41,44,60,61].

## 4. Conclusions

As a result of the conducted research, to establish an optimal and accurate model for predicting the concentration of iron in muscle tissue, it is necessary to determine the levels of magnesium, potassium, iron, aluminum, and chromium in hair (mg/kg), and perform calculations based on the compiled regression equation. The choice of model is relevant depending on the context of its use. Overall, the models presented for comparison in the study have comparable accuracy, so the choice of one over the other will depend on the conditions of animal husbandry and the capabilities for analyzing the initial samples.

In future research, there are plans to test and compare the effectiveness of the proposed models using new data to objectively assess any differences in predictive quality between the models with different predictors. To enhance the prediction accuracy, the obtained model could undergo further training. Incorporating new data into the model may necessitate the use of mixed-effects linear models, allowing for the addition of random effects and addressing multicollinearity issues and other constraints of linear models. The results obtained can be utilized in the field of ecology for assessing ecological well-being and determining the permissible iron load on animals. For veterinary medicine, the obtained model provides the opportunity for a lifelong evaluation of iron levels in Hereford cattle muscle tissue.

## Figures and Tables

**Table 1:** Designation and decoding of the set of independent variables used for selecting regression models.

Indicator	Median (Q1–Q3)	Units of Measurement	Variable in the Model
Fe muscles	22.4 (19–29.5)	mg/kg	y
P hair	250 (201.7–300)	mg/kg	x1
Ca hair	2200 (1800–2600)	mg/kg	x2
Mg hair	410 (240–655)	mg/kg	x3
Na hair	190 (99–958.3)	mg/kg	x4
K hair	83 (38.3–863.3)	mg/kg	x5
Fe hair	29 (23.2–49)	mg/kg	x6
Mn hair	22 (13.2–41.2)	mg/kg	x7
Cu hair	9.6 (7.85–12)	mg/kg	x8
Zn hair	130 (110–170)	mg/kg	x9
Al hair	20 (12–29,3)	mg/kg	x10
Ba hair	2.7 (1.5–4.8)	mg/kg	x11
Cr hair	8.2 (5.4–11)	mg/kg	x12

**Table 2:** Parameters for estimating the coefficients of the candidate model (by stepwise algorithm) for forecasting iron levels in muscle tissue (mg/kg) from blood indices.

Coefficients’ Notation	Coefficients Estimates	Standard Errors of Coefficients	t-Statistic	P_t_
Intercept	25.556	2.766	9.239	<0.001
Mg	−0.039	0.009	−4.615	0.000
Na	−0.003	0.002	−1.067	0.297
K	0.010	0.003	3.601	0.001
Fe	−0.218	0.050	−4.395	<0.001
Al	0.271	0.068	3.952	0.001
Cr	1.767	0.511	3.461	0.002
RSE—4.567; F-statistic—7.197; *p* < 0.001.				

Note: RSE: residual standard error.

**Table 3:** Parameters for evaluating the coefficients of the candidate model (Bayesian information criterion, Akaike criterion, and Mallows criterion) for predicting the level of iron in muscle tissue.

Coefficients’ Notation	Coefficients Estimates	Standard Errors of Coefficients	t-Statistic	P_t_
Intercept	25.862	2.759	9.374	<0.001
Mg	−0.043	0.008	−5.323	<0.001
K	0.008	0.002	4.201	<0.001
Fe	−0.214	0.050	−4.320	<0.001
Al	0.235	0.060	3.916	<0.001
Cr	1.904	0.496	3.841	<0.001
RSE—4.58; F-statistic—10.44; *p* < 0.001.				

Note: RSE: residual standard error.

**Table 4:** Candidate models for predicting the level of iron in muscle tissue based on internal quality criteria.

The Formula of the Model	df	*p*	SSE	MSE	R^2^	R^2^_adj_	AIC	BIC
The best model based on the R^2^_adj_ value								
y~1 + x3 + x4 + x5 + x6 + x10 + x12	24	6	500.62	20.86	0.69	0.61	190.21	220.16
The best model based on BIC, AIC, and Mallows’ criterion values								
y~1 + x3 + x5 + x6 + x10 + x12	25	5	524.38	20.98	0.68	0.61	189.65	218.17

Note: SSE: sum of squares error; MSE: mean squared error; AIC: Akaike information criterion; BIC: Bayesian information criterion; y: Fe in muscle tissue; x3: Mg in hair; x4: Na in hair; x5: K in hair, x6: Fe in hair; x10: Al in hair; x12: Cr in hair.

**Table 5:** Variance inflation factor (VIF) values for the coefficients of regression models predicting the level of iron in the muscle tissue.

Predictor	Complete Model	Fe~Mg + Na + K + Fe + Al + Cr	Fe~Mg + K + Fe + Al + Cr
P	3.7		
Ca	5.5		
Mg	11	5.1	4.5
Na	8.2	5	
K	7.3	5	2.2
Fe	2.3	1.3	1.3
Mn	2.4		
Cu	2		
Zn	3.8		
Al	2.1	1.5	1.2
Ba	3.2		
Cr	12	6.2	5.8

**Table 6:** Evaluation of error in cross-validation of regression models for predicting iron levels in muscle tissue.

The Formula of the Model	SS	df	MS	R^2^	R^2^_cv_
y~1 + x3 + x5 + x6 + x10 + x12	776.06	31	25.03	0.68	0.55
y~1 + x3 + x4 + x5 + x6 + x10 + x12	1695.66	31	54.7	0.69	0.47
y~1 + x1 + x2 + x3 + x4 + x5 + x6 + x7 + x+x9 + x10 + x11 + x12	3382.13	31	109.1	0.73	0.3

Note: R^2^_cv_ is the coefficient of determination calculated using the cross-validation method.

## Data Availability

The data presented in this study are available upon request from the corresponding author. The data are not publicly available due to ethical restrictions.
